# Combined Central Retinal Artery Occlusion (CRAO) and Central Retinal Vein Occlusion (CRVO) in a Celiac Disease Patient: A Case Report

**DOI:** 10.7759/cureus.51567

**Published:** 2024-01-03

**Authors:** Fahad Al Fardan, Mohammed H Aldebasi, Faisal Y AlThekair, Muataz Guma, Tariq Aldebasi

**Affiliations:** 1 Ophthalmology, King Abdulaziz Medical City (KAMC) Ministry of National Guard Health Affairs (MNGHA), Riyadh, SAU; 2 Ophthalmology, King Abdullah International Medical Research Center (KAIMRC), Riyadh, SAU; 3 Neurology, King Saud Bin Abdulaziz University for Health Sciences, Riyadh, SAU; 4 Neurology, King Abdullah International Medical Research Center (KAIMRC), Riyadh, SAU

**Keywords:** combined central retinal artery and vein occlosion, central retinal vein occlusion (crvo), central retinal artery occlusion, s: celiac, s: celiac disease

## Abstract

Celiac disease (CD) is a digestive disorder caused by an abnormal immune reaction to gluten, leading to severe malabsorption syndrome. Central retinal vein occlusion (CRVO) was reported in a couple of cases worldwide in patients with this disease entity. Herein, we are reporting a rare case of combined central retinal vein and artery occlusion in a young female celiac disease patient presented with a counting finger vision at six feet and improved to 20/60 vision after conservative management.

## Introduction

Celiac disease (CD) is a digestive disorder caused by an abnormal immune reaction to gluten. The latter is a protein found in foods made with wheat, rye, and other grains. In addition, gluten can be found in some medications, dietary supplements, and vitamins. It contains two major protein fractions, the gliadins and the glutenins, both of which contain disease-activating proteins. The pathogenesis of celiac disease includes environmental, immunologic, and genetic factors in people who carry the HLA class 2 gene (HLA-DQ2 or -DQ8 haplotype), who are more susceptible to acquiring the disease. These factors can lead to mucosal damage of the small intestines, resulting in malabsorption syndrome [1-2]. The intestinal villi's complete damage and total atrophy can ensue in severe cases. Malabsorption syndrome can result in iron deficiency anemia, weight loss, skin rash, thrombus formation, and other symptoms of vitamin and mineral deficiency [3].

In celiac disease, thrombosis in unusual body locations is one of the complications of non-compliance with the gluten-free diet. Few central retinal vein occlusion (CRVO) cases were reported worldwide in patients with CD [4-7]. Combined central retinal artery occlusion (CRAO) and CRVO are uncommon retinal vascular diseases causing sudden visual acuity loss [8]. In young adults, this condition is rare and usually attributed to thromboembolic disorders and vasculitis.

To the best of our knowledge, no previous reports in the literature regarding CRAO and celiac disease. Herein, we report the first case of combined central retinal artery and vein occlusion in a young patient with celiac disease.

## Case presentation

**Figure 1:**
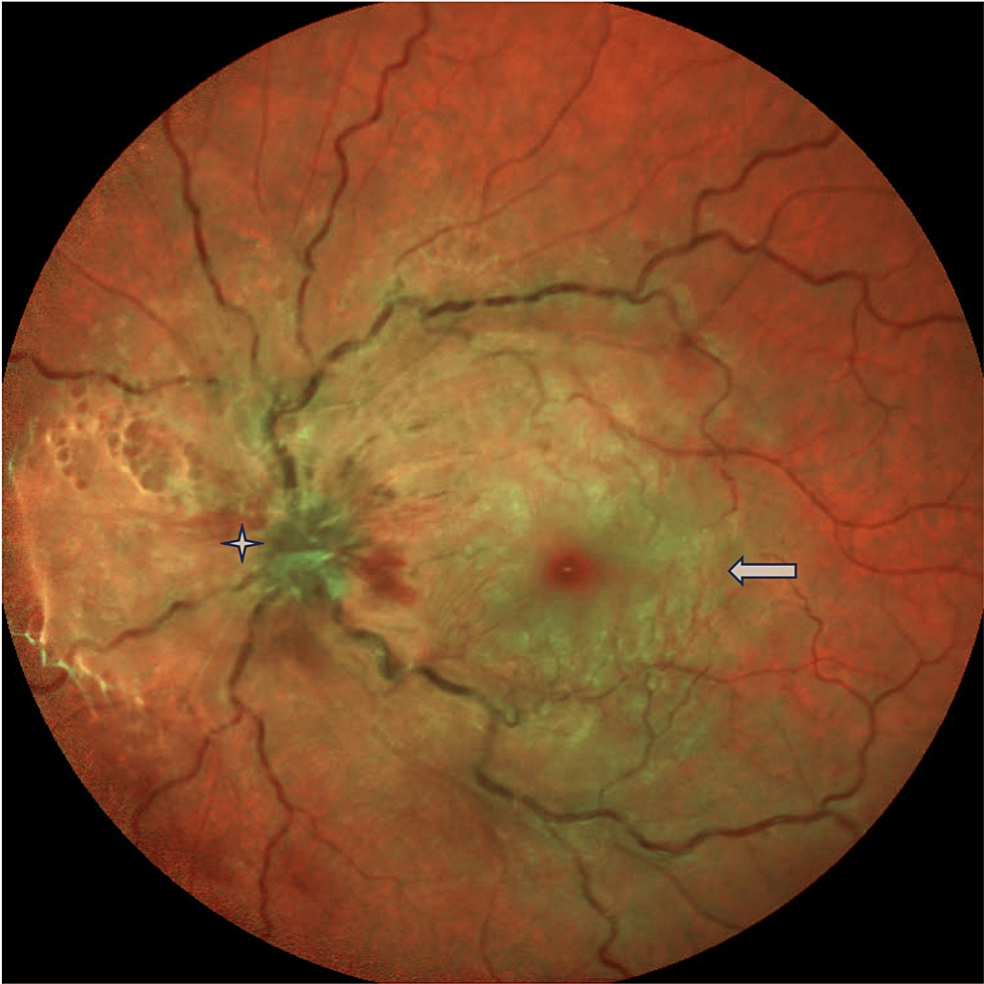
Left eye fundus photo two days after the presentation Fundus photo of the left eye showing congested veins and edematous disc with resolving cherry red spot. The arrow indicates a cherry red spot. The asterisk highlights the edematous optical disc.

**Figure 2:**
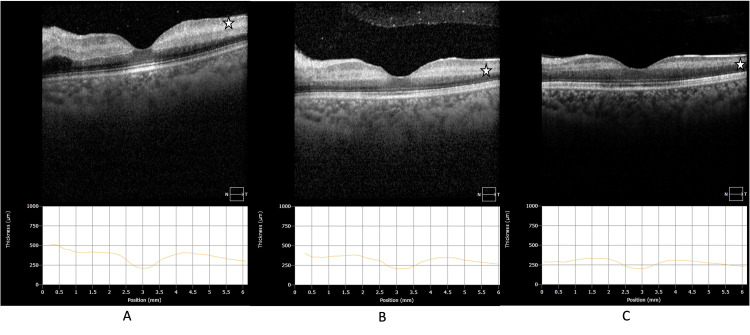
Left eye Optical Coherence Tomography (OCT) at the presentation and two weeks later Left eye Optical Coherence Tomography (OCT) (A) at the presentation, (B) after 4 days, (C) after two weeks. The asterisk in (A) shows marked retinal edema, which resolved, and atrophy developed, as highlighted by the asterisk in (C) after two weeks.

A 33-year-old woman with a known case of celiac disease for the past 5 years came to the emergency room with a sudden loss of vision in her left eye of ten hours duration. The best corrected visual acuity (BCVA) was 20/30 in the right eye, and counting fingers at 6 feet only in the left eye. We do not have a baseline visual acuity documented in the system as this is the first visit for this patient to our service. Anterior segment evaluation for both eyes was unremarkable. Intraocular pressure (IOP) was found to be 16 mmHg in the right eye and 18 mmHg in the left eye. Fundus examination was unremarkable for the right eye. On the other hand, her left eye showed marked ischemic retinal whitening with a cherry red spot, mild venous tortuosity, and hyperemic optic disc with no vitritis (Figure 1). Optical Coherence Tomography (OCT) of the left eye showed marked retinal edema initially (Figure 2).

The patient was initially diagnosed as a case of CRAO, and with a series of examinations, it was revealed that she has an impending CRVO as well. Digital massage was done initially, along with IOP lowering agents to the left eye in addition to systemic dorzolamide as part of CRAO acute care management despite her relatively late presentation. A thorough dilated fundus examination did not reveal the presence of any intra-arterial emboli.

The patient was then admitted for further workup and inpatient consultations with both internal medicine and hematology services. During her admission, the patient underwent multiple investigations, including cardiac evaluation with an echocardiogram and carotid doppler, computed tomography angiography (CTA), and brain magnetic resonance imaging (MRI) with contrast, which were all unremarkable, except for low level of hemoglobin 58 gm/l (120-160 gm/l), prothrombin time (PT) 14.60 (9.38-12.34), INR 1.35 (0.8-1.2 seconds). Infectious serology to exclude infectious causes was done, including syphilis, cytomegalovirus, and tuberculosis, which was negative. 

**Figure 3:**
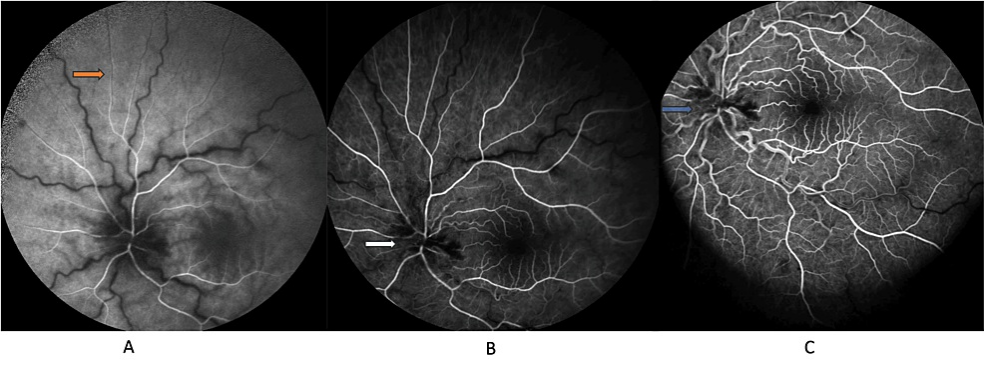
Fundus fluorescein angiography (FFA) of the left eye two days from the presentation Fundus fluorescein angiography (FFA) photo montage throughout the study. (A) and (B) showing arteriolar narrowing, (C) highlighting dilated veins and delayed venous filling.

Two days later, the picture of CRVO became more evident in the form of edematous disc and retinal hemorrhages in all four quadrants. At that time, CRAO was aborted with the fading of the cherry red spot. Also, fundus fluorescein angiography (FFA) revealed a patent retinal artery with mild narrowing of retinal arterioles and no retinal arterial filling delay, which supports the diagnosis of aborted CRAO. Dilated veins with delayed venous filling were observed, which confirmed the picture of CRVO (figure 3).

**Figure 4:**
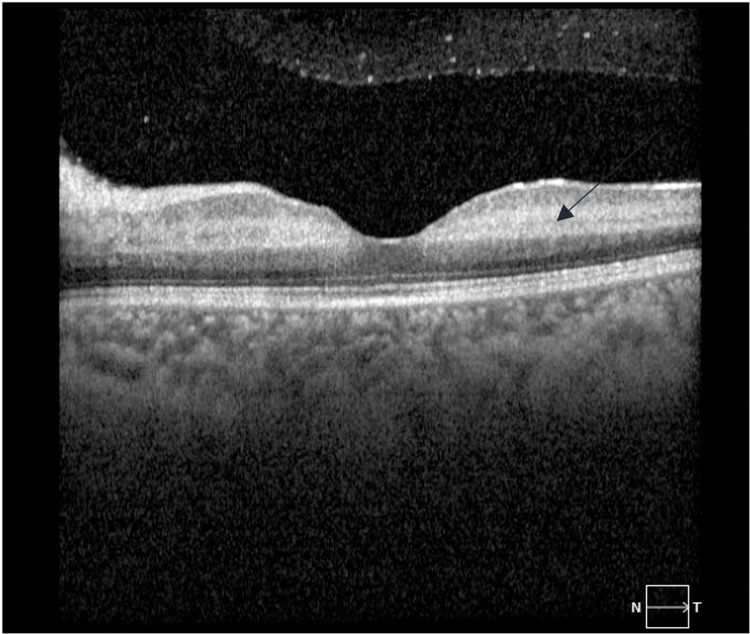
Left eye optical coherence tomography (OCT) after ten days of gluten-free diet Left eye optical coherence tomography (OCT) after ten days of gluten-free diet showing early signs of retinal atrophy (arrow).

After ten days of a strict gluten-free diet and supplemental therapy with folate, vitamin B12, vitamin D, and iron significantly improved their malabsorption status, which reflected positively on her retinal examination, and her visual acuity improved from counting fingers to 20/60 on the Snellen chart testing. OCT was done and showed resolved retinal edema with early signs of atrophic changes (Figure 4).

## Discussion

Venous thrombosis is one of the possible presentations of celiac disease. Many mechanisms were postulated for retinal vein occlusion in the literature, including vitamin K absorption insufficiency and folate deficiencies, vitamin B12, protein S, and protein C. In addition, hyperhomocysteinemia and dehydration with hyperviscosity due to malabsorption and chronic diarrhea [2-3,9].

Arterial occlusion was not reported before in celiac disease patients. The medical assessment of this young female did not reveal any positive risk factors for arterial occlusion. Her cardiac investigation and blood workup were negative. The most likely reason for her CRAO is hypercoagulability, secondary to dehydration.

The patient was first diagnosed with celiac disease in 2018, as confirmed by a duodenal biopsy. Moreover, she was educated about the importance of a gluten-free diet. Unfortunately, she was not compliant with her gluten-free diet at the time of her presentation.

Ocular examination was consistent with acute CRAO with impending CRVO, which became prominent with an edematous disc and venous congestion two days later. Initiation of intravenous fluid, ocular massage, and antiglaucoma agents possibly helped to abort CRAO and re-establish retinal artery perfusion. Typically, CRAO in young patients is the result of non-arteritic occlusion by a platelet-fibrin thrombus, embolic CRAO from an atherosclerotic lesion or secondary to a hypercoagulable state similar to the case of celiac disease [10].

Giant cell arteritis was unlikely, considering the patient's age, normal erythrocyte sedimentation rate (ESR), and C-reactive protein (CRP) levels. Additionally, the patient was promptly evaluated, and an MRI of the brain with contrast was obtained, which was unremarkable for any acute infarction or blood clots.

Combined cases of CRAO and CRVO are uncommon, tend to cause sudden visual acuity loss, and are associated with poor visual prognosis [6]. Unlike in this case, where a strict gluten-free diet and supplemental therapy with intravenous fluid, folate, vitamin B12, vitamin D, and iron significantly improved her malabsorption status, reflecting positively on her retinal examination and vision.

## Conclusions

In conclusion, combined cases of CRAO and CRVO are sight-threatening diseases that can lead to permanent visual loss. This mandates abrupt diagnosis and early management with the identification of the primary cause. All efforts should be made to minimize related morbidity. Celiac disease patients tend to have dehydration and subsequent hypercoagulable states that might yield combined CRVO and CRAO, as in our case. The importance of a multidisciplinary team approach in atypical cases can not be overstated. Celiac disease as a cause of combined CRAO and CRVO might have a better prognosis than other etiologies.
